# Cyanamide is biosynthesized from l-canavanine in plants

**DOI:** 10.1038/srep10527

**Published:** 2015-05-27

**Authors:** Tsunashi Kamo, Sakae Sakurai, Tatsuya Yamanashi, Yasushi Todoroki

**Affiliations:** 1 Biodiversity Division, National Institute for Agro-Environmental Sciences, 3-1-3 Kan-nondai, Tsukuba, 305-8604 Ibaraki, Japan; 2 Graduate School of Agriculture, Shizuoka University, 836 Ohya, Suruga-ku, 422-8529 Shizuoka, Japan; 3 Faculty of Agriculture, Shizuoka University, 836 Ohya, Suruga-ku, 422-8529 Shizuoka, Japan; 4 Research Institute of Green Science and Technology, Shizuoka University, 836 Ohya, Suruga-ku, 422-8529 Shizuoka, Japan

## Abstract

Cyanamide had long been recognized as a synthetic compound but more recently has been found as a natural product from several leguminous plants. This compound’s biosynthetic pathway, as yet unelaborated, has attracted attention because of its utility in many domains, such as agriculture, chemistry, and medicine. We noticed that the distribution of L-canavanine in the plant kingdom appeared to include that of cyanamide and that the guanidino group structure in L-canavanine contained the cyanamide skeleton. Here, quantification of these compounds in *Vicia* species suggested that cyanamide was biosynthesized from L-canavanine. Subsequent experiments involving L-[guanidineimino-^15^N_2_]canavanine addition to young *Vicia villosa* seedlings resulted in significant incorporation of ^15^N-label into cyanamide, verifying its presumed biosynthetic pathway.

Cyanamide (NH_2_CN) was first synthesized from ammonia and cyanogen chloride by Cannizzaro and Cloëz in 1851^1^. The scheme for its mass production as a calcium salt was established in Germany in the late 19th century. Cyanamide is multifunctional for agricultural purposes because it serves in the soil as an insecticide, fungicide, and herbicide for a time after application, then decomposes to urea in the soil, and finally is absorbed by crops as fertilizer. Maier-Greiner *et al.* isolated and characterized cyanamide hydratase from a soil fungus *Myrothecium verrucaria*^2^,^3^. The high specificity of this enzyme was surprising because the substrate had not been previously found in nature^3^. In 2003, natural cyanamide was first isolated from hairy vetch, *Vicia villosa*^4^. The distribution of this compound in the plant kingdom seems highly limited, having only been detected in four leguminous species among more than 550 species tested^5^,^6^. Several studies have suggested that cyanamide in plants might function in the context of chemical ecology; for example, livestock feeding on vetch-dominant meadows often die of vetch-disease^7^,^8^,^9^, probably due to cyanamide toxicity^6^,^10^.

L-Canavanine is the guanidinooxy structural analogue of L-arginine. When assimilated, it is used for peptide extension in place of L-arginine, resulting in protein malfunction^11^,^12^, which explains its toxicity to a wide range of organisms^13^. A variety of leguminous species accumulate this toxic compound in their seeds for protection from herbivores as well as nitrogen storage^14^,^15^. L-Canavanine distribution in the plant kingdom is broader than cyanamide; it has been found in more than 1500 members of the Faboideae, a subfamily of the Fabaceae^16^,^17^.

L-Canavanine’s biosynthetic pathway has already been established and described in jack bean to involve the conversion of L-canavaninosuccinic acid to L-canavanine and fumaric acid^18^. In contrast, cyanamide biosynthesis has remained totally undescribed. Although there have been no reports that indicate a biosynthetic relationship between L-canavanine and cyanamide, we focused here on the following information: First, canavanine has been detected in many *Vicia* species^16^, some of which also contain cyanamide^5^,^6^. Second, L-canavanine’s chemical structure consists of cyanamide and another amino acid, L-canaline, and it is possible to actually synthesize L-canavanine from them in the presence of zinc cations^19^. And third, L-canavanine is stored in seeds, whereas cyanamide starts to accumulate after germination^6^. Thus, it is conceivable that in plants L-canavanine is the biosynthetic precursor of cyanamide. In the present study, we conducted several experiments to examine the validity of this hypothesis.

## Results and Discussion

**Figure 1:**
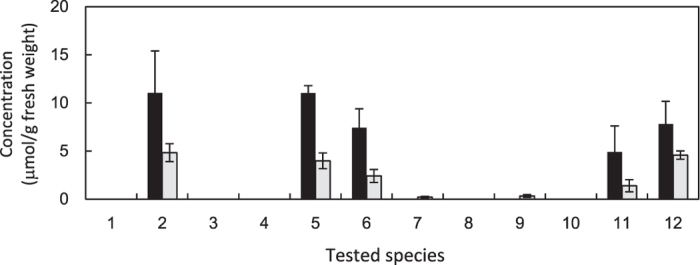
Concentration of cyanamide and L-canavanine in the leaves of leguminous plants. Tested species with sampling day after sowing in parentheses; 1, *Lens culinaris* (11 d); 2, *Robinia pseudoacacia* (22 d); 3, *Vicia angustifolia* (11 d); 4, *V. amoena* (18 d); 5, *V. benghalensis* (10 d); 6, *V. cracca* (14 d); 7, *V. hirsuta* (11 d); 8, *V. pseudo-orobus* (22 d); 9, *V. tetrasperma* (13 d); 10, *V. unijuga* (18 d); 11, *V. villosa* subsp. *villosa* (10 d); and 12, *V. villosa* subsp. *varia* (8 d). First, second and third leaves sampled when third leaf developed; black and grey bars, cyanamide and L-canavanine, respectively; and error bars, standard deviation (n = 4).

We quantified cyanamide and L-canavanine in the leaves of leguminous plants (Fig. 1). In addition to *Vicia* species, two other species were also analyzed; *Robinia pseudoacacia* as a lone species so far tested that contains cyanamide other than *Vicia* species^5^, and *Lens culinaris* for its placement, by recent molecular phylogeny studies, within a monophyletic group of the genus *Vicia*^20^,^21^. Notably, L-canavanine has been detected in all species that contain cyanamide, supporting the idea that there is a link between cyanamide biosynthesis and L-canavanine.

**Figure 2:**
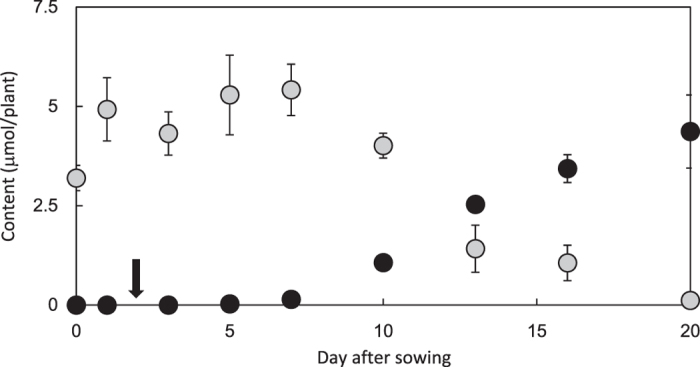
Changes in cyanamide and L-canavanine content in *V. villosa* subsp. *varia* seedlings. Black and grey circles, cyanamide and L-canavanine, respectively; arrow, germination; and error bars, standard error (n = 6).

Figure 2 presents the changes in cyanamide and L-canavanine content in young seedlings of *V. villosa* subsp. *varia*. Cyanamide was absent in the seed and began to accumulate when the first leaf appeared at 5–6 day after sowing. In contrast, L-canavanine was abundant in the seed and started to decrease after leaves developed. This synchrony was thought-provoking, particularly with the intriguing observation that the total molar content of the two compounds per individual plant remained nearly constant throughout the monitored period. This suggested that L-canavanine was a cyanamide biosynthetic precursor. It is unclear whether L-canavanine is biosynthesized at the early stage of development.

The plant organ in which cyanamide was biosynthesized was investigated by quantifying the two compounds in each part of the seedling: cotyledon, root, epicotyl, and leaf (Table S1). Since *V. villosa* is a plant with hypogeal germination, the cotyledons stay below the ground. More than half of the total L-canavanine was present in the cotyledons, which was a reasonable localization because it is contained in the seeds (Fig. 2). Nearly 40% of the total L-canavanine, probably transferred from the cotyledons, was present in the epicotyl and leaves. In contrast, almost all the cyanamide was detected in the epicotyl and leaves. Considering that this compound started to accumulate when leaves emerged, cyanamide appeared to be biosynthesized in leaves. Thus, it was highly likely that L-canavanine stored in the seed was transported to the seedling shoot after germination and then converted to cyanamide in leaves.

**Figure 3:**
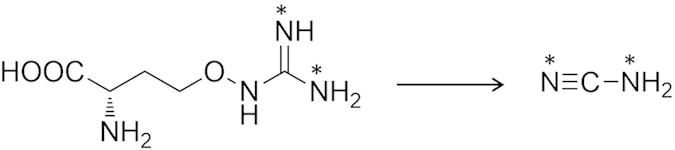
Cyanamide biosynthesis from L-canavanine.

We confirmed the conversion of L-canavanine to cyanamide by administration of ^15^N-labelled compounds to 4-day-old shoots of *V. villosa* subsp. *varia*. After 48 h of incubation, cyanamide was isolated from the leaves and analyzed by gas chromatography-mass spectrometer (GC-MS). Compared with the control, the isotopic molecular ion peak at *m/z* 44 (the [M + 2]^+^ ion) was clearly enhanced when L-[guanidineimino-^15^N_2_]canavanine was administered (Table 1; Figures S1 and S2). This indicated that this substrate was converted to [^15^N_2_]cyanamide in shoots (Fig. 3). Administration of [^15^N_2_]ammonium nitrate and [^15^N_2_]urea showed no effect on the abundance of key isotopic molecular ion peaks, negating the possibility that L-canavanine degradative products, such as urea or ammonia, were used for cyanamide biosynthesis. ^15^N-Label from L-[guanidineimino-^15^N_2_]arginine was also not incorporated into cyanamide, which illustrated a clear difference in incorporation between L-canavanine and L-arginine and implied high substrate specificity by the enzyme responsible for cyanamide production.

Plant arginase can convert L-canavanine into L-canaline and urea, but L-arginine is a better substrate for this enzyme (Figure S3)^22^,^23^. In addition to this conversion, L-canavanine is also decomposed to smaller molecules or coupled with other compounds by enzymatic reactions in which L-arginine is the favored primary substrate^24^. In contrast, some canavanine-resistant microorganisms benefit from mechanisms that specifically function on this toxic amino acid; it is cleaved to L-homoserine and guanidine in *Streptococcus faecalis* and *S. equinus* and hydrolyzed to L-homoserine and hydroxyguanidine in *Pseudomonas* sp. (Figure S4)^24^,^25^. The former route reportedly also functions in canavanine-resistant insects, such as the tobacco budworm *Heliothis virescens*^26^. Our results here indicated that a novel enzymatic activity specific for L-canavanine was present in some *Vicia* species. Although the amino acid formed in this cleavage is yet to be clarified, the conversion from L-canavanine to cyanamide is reasonably explainable by a general acid-base catalysis mechanism (Figure S5).

In conclusion, we demonstrated that cyanamide was biosynthesized from L-canavanine in plants. As cyanamide has a short history since it was first isolated from a natural source^4^, the biosynthesis and metabolism of natural cyanamide has not been fully explained. This finding will serve, in future studies, as a crucial step for the isolation and characterization of the responsible enzyme.

## Methods

### General

GC-MS was performed on a QP5050A system (Shimadzu Corp., Kyoto, Japan) using an Equity-5 column (0.25 mm i.d. × 30 m, 0.25 μm film thickness; Supelco, Inc., Bellefonte, PA, USA). The HPLC consisted of a 626 pump with 996 photodiode array detector (Waters Corp., Milford, MA) equipped with a C-18 column (Inertsil ODS-3, 5 μm, 250 × 4.6 mm ID; GL Sciences, Inc., Tokyo, Japan). ^1^H and ^13^C NMR spectra were recorded with tetramethylsilane as the internal standard using JNM-EX270 (270 MHz) and JNM-LA500 (500 MHz) NMR spectrometers (JEOL Ltd., Tokyo, Japan). High resolution mass spectra were obtained with a JMS-T100LC AccuTOF mass spectrometer (JEOL). The seeds used in the present study have been previously described^6^. They were planted at a depth of 5 mm in pots (8 × 8 × 6 cm deep) containing sand and then incubated in an illuminated growth chamber (FLI-301N; Tokyo, Rikakikai, Co., Ltd., Tokyo, Japan) under a 16-h light/8-h dark cycle at 22 °C.

### ^15^N-Labelled chemicals

[^15^N_2_]Urea (98+ atom% ^15^N) was purchased from Sigma-Aldrich Corp. (Milwaukee, WI, USA). L-[Guanidineimino-^15^N_2_]arginine hydrochloride (min 98 atom% ^15^N), [^15^N_2_]ammonium nitrate (min 98+ atom% ^15^NH_4_, min 98+ atom% ^15^NO_3_) and [^13^C,^15^N_2_]cyanamide (99 atom % ^13^C, 98 atom % ^15^N, 50 wt% aqueous solution) were purchased from Isotec (Miamisburg, OH, USA). L-[Guanidineimino-^15^N_2_]canavanine was synthesized from [^15^N_2_]urea (Figure S6).

### Quantification of cyanamide and L-canavanine in leguminous species

Fresh plant materials (20–50 mg fresh weight) were extracted with 1 mL ethanol, according to a published procedure^6^, and the extracts used for quantification of cyanamide and L-canavanine. The leaves obtained from a single seedling was used for one lot of the extraction. For cyanamide quantification by GC-MS, 50 μL of [^13^C,^15^N_2_]cyanamide solution (10 μg/mL in acetonitrile) was added as internal standard to the ethanol extract (50 μL; GC-MS conditions^6^). For L-canavanine quantification by HPLC, the ethanol extract (160 μL) was mixed with 20 μL triethylamine and 20 μL phenyl isothiocyanate and then placed at room temperature for 20 min before concentrating the solution to dryness in vacuo^27^,^28^. The residue was dissolved in 100 μL ethanol, and a 10-μL aliquot injected into the HPLC (HPLC conditions^29^). For cyanamide and L-canavanine quantification in 12 leguminous species (Fig. 1), leaves were sampled when the third leaf developed. The species used are listed here with the sampling day after sowing in parentheses: *Lens culinaris* (11 d), *Robinia pseudoacacia* (22 d), *Vicia angustifolia* (11 d), *V. amoena* (18 d), *V. benghalensis* (10 d), *V. cracca* (14 d), *V. hirsuta* (11 d), *V. pseudo-orobus* (22 d), *V. tetrasperma* (13 d), *V. unijuga* (18 d), *V. villosa* subsp. *villosa* (10 d), and *V. villosa* subsp. *varia* (8 d).

### Synthesis of L-[guanidineimino-^15^N_2_]canavanine

L-Canaline was prepared using a modified previously-described method^30^. A solution of benzyl L-2-[(carbobenzyloxy)amino]-4-(benzamidooxy)butanoate (1.26 g, 2.72 mmol) in 4 M HCl (30 mL) was refluxed for 5 h and the solvent removed in vacuo. The residue was dissolved in ethanol (1.5 mL), precipitated with diethyl ether (6 mL) and filtered. The resultant white cake was washed with diethyl ether (2 × 6 mL) and dissolved in ethanol (1.5 mL). Triethylamine (10 mL) was added to produce a white precipitate, which was concentrated by solvent removal in vacuo. The residue was dissolved in water (15 mL), charcoal added, and the mixture stirred for 10 min. Next, it was filtered and concentrated in vacuo to yield a white solid (0.99 g), which was then washed with ethanol (2 × 10 mL) to remove the triethylamine hydrochloride. The residual oil containing a white solid was purified by silica gel column chromatography with ethanol/water stepwise to obtain L-canaline (177 mg, 1.32 mmol, 49% yield) as a colorless oil. ^1^H NMR (270 MHz, D_2_O): δ 3.69 (m, 3H), 2.00 (m, 2H); HRMS (*m*/*z*): [M-H]^−^ calcd. for C_4_H_9_N_2_O_3_, 133.0613; found, 133.0609 (ESI-TOF, negative mode).

L-[Guanidineimino-^15^N_2_]canavanine was prepared from L-canaline and [^15^N_2_]-*O*-methylisourea methylsulfate by a modified previously-described method^31^,^32^. [^15^N_2_]-*O*-Methylisourea methylsulfate was obtained from [^15^N_2_]urea using the same method as for preparation of the non-labelled compound^33^. Cupric oxide (239 mg) was added to a stirred solution of L-canaline (177 mg, 1.32 mmol) in water (2 mL) and the solution refluxed for 10 min to obtain a copper/L-canaline complex. The resultant deep blue solution was next stirred at 60 °C for 2 h and at room temperature for 4 d. After filtration, the filtrate was concentrated to a small volume (~2 mL) in vacuo and then [^15^N_2_]-*O*-methylisourea methylsulfate (440 mg, 2.30 mmol) dissolved in water (1.2 mL) added at 0 °C. After addition of 2 M NaOH aq. (1.2 mL), the solution was stirred for 16 d at room temperature, acidified (pH ~1) with 1 M HCl (3.6 mL) to pale blue, and treated with hydrogen sulfide. After filtration to remove the resulting black precipitate (CuS), the filtrate was heated at 90 °C for 5 h to remove hydrogen sulfide and concentrated in vacuo to produce a crude oil containing a pale yellow solid (603 mg). Flavianic acid dehydrate (460 mg, 1.31 mmol) in water (0.6 mL) was added to the crude product in water (3 mL) and held at 5 °C for 17 h. After suction filtration, the resulting yellow crystals were collected and dissolved in water (10 mL) at ~90 °C. Barium hydroxide octahydrate (310 mg) dissolved in hot water (10 mL) was added to the solution, which was held at 5 °C for 16 h and then filtered. The filtrate was next treated with 5% sulfuric acid (0.6 mL) and filtered through filter paper to remove the white precipitate. The yellow filtrate was then treated with charcoal for 30 min, filtered, and concentrated in vacuo. The residual colorless oil (48 mg) was purified by strong cation exchange resin column chromatography (PoraPak Rxn CX, Waters Corp., Milford, MA, USA) with 5% 3 M ammonia solution in methanol. The eluate was concentrated in vacuo and recrystallized in ethanol/water (25/1, v/v) to obtain L-[guanidineimino-^15^N_2_]canavanine (2.0 mg, 11 μmol, 0.9% yield). ^1^H NMR (500 MHz, D_2_O): δ 3.83 (m, 2H), 3.73 (t, *J* = 5.8 Hz, 1H), 2.13 (m, 1H), 2.06 (m, 1H); ^13^C NMR (125 MHz, D_2_O): δ 176.2, 159.8, 70.9, 54.4, 31.2; HRMS (*m*/*z*): [M + H]^+^ calcd. for C_5_H_13_N_2_^15^N_2_O_3_, 179.0928; found, 179.0923 (ESI-TOF, positive mode).

### Administration of ^15^N-labelled compounds to *Vicia villosa* subsp. *varia* seedlings

A 4-day-old *V. villosa* subsp. *varia* shoot (leaves plus epicotyl) excised from the roots and cotyledons was inserted into each tube containing 0.8 mL of a solution of ^15^N-labelled compound (2.0 mM). The shoots were next incubated for 48 h in the illuminated growth chamber under a 16-h light/8-h dark cycle at 22 °C, with the solution replaced at 24 h. The leaves (~30 mg fresh weight) were used for cyanamide analysis (for the extraction procedure, see the literature^6^). Out of 900 μL of ethanol extract, a 300 μL aliquot was used for purification. After the addition of 60 μL of water, the ethanol extract was concentrated in vacuo and 20 μL of the resulting water solution (ca. 30 μL) then purified by HPLC to yield a cyanamide-containing fraction (*t*_R_ 3.3–3.9 min), which was then concentrated to dryness and dissolved in 50 μL ethyl acetate. A portion of the sample solution was finally subjected to GC-MS, using previously described conditions^6^, except the splitless injection mode was used with a 2 min sampling time at 200 kPa, and the sample injection volume was at 2.5 μL.

## Additional Information

**How to cite this article**: Kamo, T. *et al.* Cyanamide is biosynthesized from L-canavanine in plants. *Sci. Rep.* **5**, 10527; doi: 10.1038/srep10527 (2015).

## Supplementary Material

Supplementary Information

## Figures and Tables

**Table 1:** Incorporation of ^15^N-labels into cyanamide in shoots of 4-day-old seedlings of *V. villosa* subsp. *varia*.

**Treatment**	Relative area of the isotopic [M]^+^ ions at *m/z* 42 to 45 (%)*			
	**42**	**43**	**44**	**45**
(Theoretical value)	100.0	1.9	0.0	0.0
Control	100.0	1.9	0.1	0.0
[^15^N_2_]Ammonium nitrate	100.0	1.7	0.1	0.0
[^15^N_2_]Urea	100.0	1.8	0.1	0.0
L-[Guanidineimino-^15^N_2_]arginine	100.0	1.9	0.1	0.0
L-[Guanidineimino-^15^N_2_]canavanine	100.0	15.1	98.4	0.8

Labelled compounds administered at 2.0 mM for 48 h to *V. villosa* shoot (leaves plus epicotyl) excised from roots and cotyledons.

^*^Cyanamide isolated from leaves analyzed by GC-MS after preparative HPLC.
