# Supplementary material for: Cyanamide is biosynthesized from l-canavanine in plants
Source: Sci Rep. 2015 May 27;5:10527. doi: 10.1038/srep10527 (PMC4650597; doi:10.1038/srep10527)
Supplement: Supplementary Information [file srep10527-s1.pdf]

Cyanamide is biosynthesized from L-canavanine in plants

Tsunashi Kamo, Sakae Sakurai, Tatsuya Yamanashi
& Yasushi Todoroki

Table S1. Cyanamide and L-canavanine distribution in 6-day-old seedlings of *V. villosa* subsp. *varia*.

	Cotyledon	Root	Epicotyl	Leaf
Fresh weight*	73.3 ±11.1	58.4 ±15.6	68.0 ±8.7	30.8 ±5.2
Cyanamide**	0.05 ±0.02	0.06 ±0.02	2.4 ±0.5	6.8 ±2.1
L-Canavanine**	48.7 ±25.9	9.2 ±4.2	18.5 ±3.7	31.2 ±17.0

Values, mean ±standard deviation (n=4). *Mass in mg. **Mass in μmol/g fresh weight.

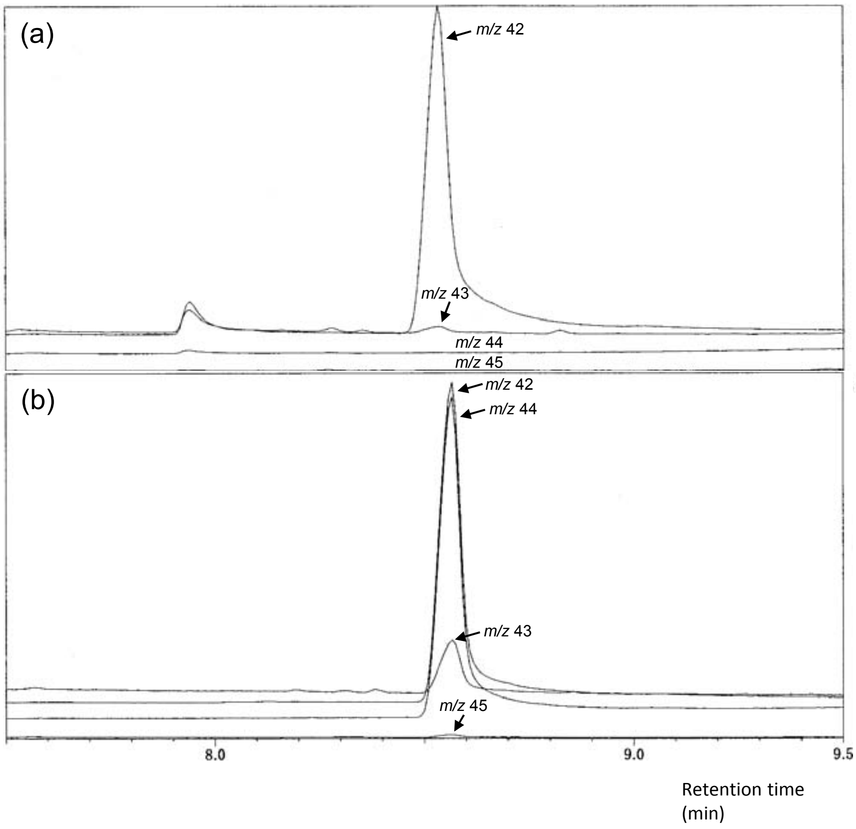

Figure S1. Mass chromatograms of cyanamide isolated from the *V. villosa* subsp. *varia* shoots. (a) Control (treatment with water). (b) Treatment with 2.0 mM of L-[guanidineimino-$^{15}N_2$]canavanine. The 4-day-old shoots were incubated for 48 h. Mass spectra were measured in selected-ion-monitored (SIM) mode (*m/z* 42, 43, 44 and 45).

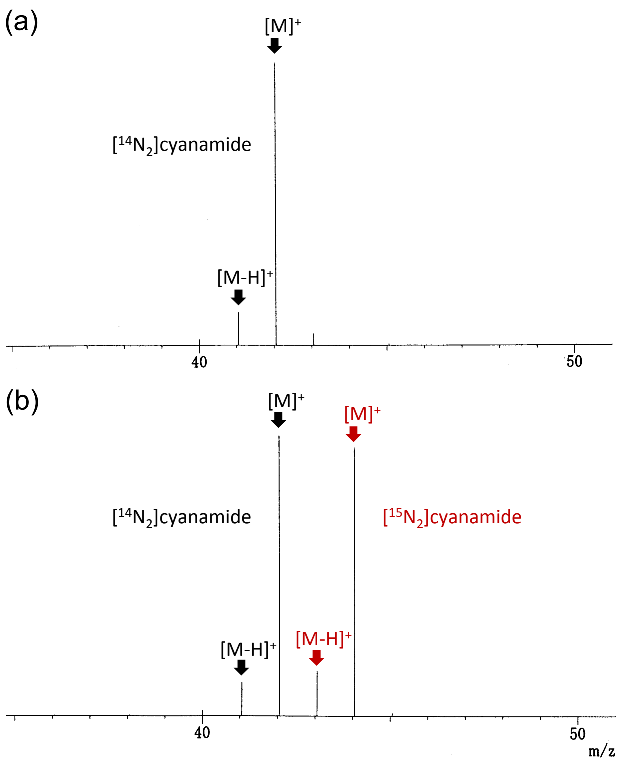

Figure S2. Mass spectra of cyanamide isolated from the *V. villosa* subsp. *varia* shoots. (a) Control (treatment with water). (b) Treatment with 2.0 mM of L-[guanidineimino-$^{15}N_2$]canavanine. The 4-day-old shoots were incubated for 48 h. Mass spectra were measured in scan mode (*m/z* 35–50). The peak at *m/z* 43 is assignable to the $[M\text{-}H]^+$ ion of [$^{15}N_2$]cyanamide and in part the natural isotopic $[M]^+$ ion of cyanamide.

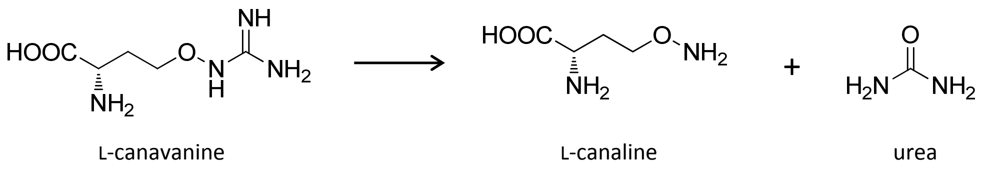

Figure S3. Metabolism of L-canavanine in plants.

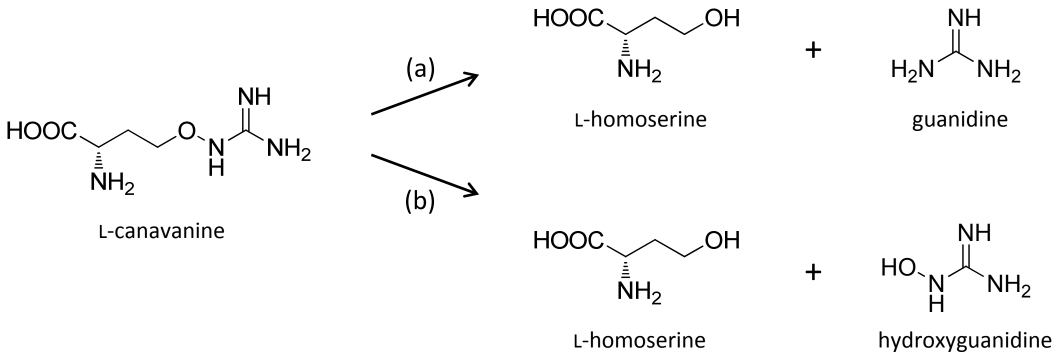

Figure S4. Metabolism of L-canavanine in microorganisms. (a) *Streptococcus feacalis* and *S. equinus*. (b) *Pseudomonas* sp.

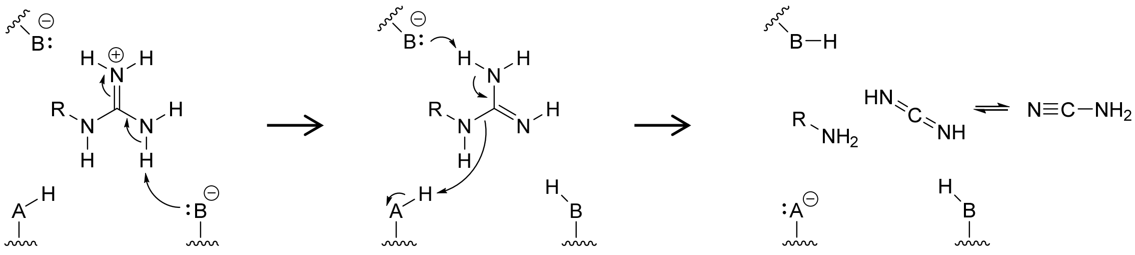

Figure S5. A general acid-base catalysis mechanism from L-canavanine to cyanamide.

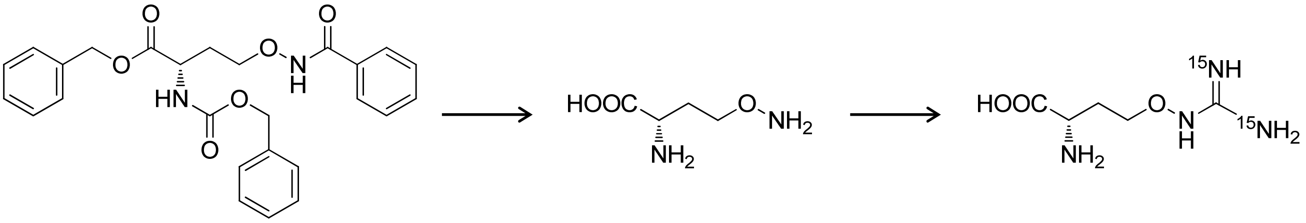

L-2-[(carbobenzyloxy)amino]-4-(benzamidooxy)butanoate

L-canaline

L-[guanidineimino-$^{15}N_2$]canavanine

Figure S6. Synthesis of L-[guanidineimino-$^{15}N_2$]canavanine.
